# Prevalence of Undernutrition and Associated Factors among Pregnant Women in a Public General Hospital, Tigray, Northern Ethiopia: A Cross-Sectional Study Design

**DOI:** 10.1155/2020/2736536

**Published:** 2020-10-07

**Authors:** Ebud Ayele, Guesh Gebreayezgi, Teklewoini Mariye, Degena Bahrey, Gebrekiros Aregawi, Gebregziabher Kidanemariam

**Affiliations:** ^1^Department of Public Health Nutrition, College of Medicine and Health Sciences, Aksum University, Aksum, Ethiopia; ^2^Department of Nursing, College of Medicine and Health Science, Aksum University, Aksum, Ethiopia; ^3^Department of Midwifery, College of Medicine and Health Science, Aksum University, Aksum, Ethiopia

## Abstract

**Background:**

Undernutrition is a global health problem, particularly in pregnant women. Despite the limited studies performed in different parts of Ethiopia, the information about the prevalence of undernutrition of pregnant women in the current study area is not documented. Therefore, this study aimed to assess the prevalence of undernutrition and associated factors in pregnant women.

**Methods:**

An institution-based cross-sectional study design was conducted in the Tigray region from August 01 to December 30, 2018. Study subjects were selected by systematic sampling technique from the respective hospitals. An interviewer-administered questionnaire was used to collect the data. Data were cleaned and entered using Epi-Data version 3.1 and then exported to statistical package for social science (SPSS) version 23.0 for analysis. Multivariate analyses were carried out, and adjusted odds ratios (AORs) with 95% CI and significance level (*p* value) <0.05 were considered.

**Results:**

Out of the total 844 selected pregnant women, 840 participated in the study, yielding a response rate of 99.5%; of this, respondent's undernutrition prevalence was found to be 40.6% with 95% confidence interval (38.93% and 42.27%). Agriculture as occupation (AOR = 2.6, 95% CI: 1.5, 4.5), women who wanted the pregnancy (AOR = 0.25, 95% CI: 0.14, 0.448), no history malaria during pregnancy (AOR = 0.291, 95%: (0.152, 0.555)), coffee intake during pregnancy (AOR = 1.6, 95% CI: 1.04, 2.69), and hemoglobin < 11 g/dl (AOR = 4.9, 95% CI: 3.09, 7.8) were the factors that were significantly associated with undernutrition, *p* value (<0.05).

**Conclusion:**

In this study, occupation, history of having malaria during pregnancy, wanted type pregnancy, coffee intake during pregnancy, and hemoglobin < 11 g/dl were factors significantly associated with undernutrition in pregnant mothers. So, healthcare providers, policymakers, and other stakeholders should give special focus on these factors.

## 1. Introduction

Pregnancy strongly depends on the health and nutritional status of women, and a high proportion of pregnant women are affected by poor nutrition which leads them to unhealthy and distress conditions [1]. Optimal nutrition in mothers is not only crucial for their health but also for the health of future generations [2]. Undernutrition is among the most common causes of maternal mortality [3, 4].

Nutrition-related problems form the core of many current issues in women's health, and poor nutrition can have profound effects on reproductive outcomes [1]. Nutrition plays a vital role in reducing some of the health risks associated with pregnancy such as the risk of fetal and infant mortality, intrauterine growth retardation, low birthweight, premature births, decreased birth defects, cretinism, poor brain development, and risk of infection [5].

The global prevalence of undernutrition in mothers is about 462 million [6, 7]. And, this becomes the most important risk factor for morbidity and mortality, and hundreds of millions of pregnant women and young children are affected by undernutrition [8]. Undernutrition is a significant global health concern, particularly in pregnant women [9]. Adequate nutrition is essential for a woman throughout her life cycle, and pregnant women require varied diets and increased nutrient intake to cope with the extra needs during pregnancy. The use of dietary supplements and fortified foods should be encouraged for pregnant women to ensure an adequate supply of nutrients for both mother and fetus [5].

If adolescents or women are undernourished during pregnancy, the cycle of maternal malnutrition, fetal growth restriction, child stunting, subsequent lifetime of impaired productivity, and increased maternal and fetal morbidity and mortality are perpetuated [10]. Pregnancy is an anabolic process, and a woman's normal nutritional requirement increases during pregnancy to meet the needs of the growing fetus and the maternal tissues associated with pregnancy. Thus, the nutritional status of the expectant mother is one of the most important determinants affecting pregnancy outcomes [11].

Therefore, to address maternal undernutrition through intervention, a scientific study was of paramount importance to assess the present image of undernutrition and to identify factors that are affecting for undernutrition, and the available pieces of literature in Ethiopian were limited in addressing factories that influence undernutrition among pregnancy mothers. Therefore, this study aims to assess the prevalence and factors associated with undernutrition among pregnant women in a public general hospital of Tigray, Northern Ethiopia.

## 2. Methods and Materials

### 2.1. Study Design, Area, and Period

An institution-based cross-sectional study design was carried out. This study was conducted in public general hospitals of the Tigray region; in the region, there were 14 total public general hospitals from those five hospitals: Mekelle public general Hospital found in the regional administration, St. Marry public general hospital found in the central zone of the region, Lemlem Karl public general Hospital found in the southern zone of the region, Kahsay Abera public general Hospital west zone of the region, and Adigrat public general hospital found in the southeast were the selected study area. Data collection for this study was undertaken from August 01 to December 30, 2018.

### 2.2. Source and Study Population

The source populations were all third-trimester pregnant women who were coming for delivery and antenatal care visits in the selected public general hospitals of the Tigray region. Third-trimester pregnancy women who were coming for delivery and antenatal care visits in general public hospitals of the Tigray region were selected as the study population.

### 2.3. Inclusion and Exclusion Criteria

All selected third-trimester pregnant women who were coming for delivery and ANC in public general hospitals during the study period were included, whereas pregnancy women with bilateral edema were excluded.

### 2.4. Sample Size and Sampling Techniques

Sample size was calculated using single population proportion formula by assuming precision (*d*) = 5%, confidence level = 95% (*Ζα*/2 = 1.96), and proportion of undernutrition (*P*) = 50%. By considering a 10% nonresponse rate, it becomes 422. Finally, 844 pregnant women were taken as a final sample size after using the design effect two. Two-stage sampling was employed to select the study participants. In Tigray, there were 14 public general hospitals; from those, five hospitals were selected randomly and the sample size was proportionally allocated to each hospital. A systematic random sampling technique was used to select every (determined interval *K* = 2) study subjects from all the five hospitals.

### 2.5. Data Collection Procedures

A semistructured questionnaire was initially prepared in English and then translated into the local language; Tigrigna was used. Tigrigna version was again translated back to English to check for any inconsistencies or distortion in the meaning of words. Data were collected using an interviewer-administered, and MUAC measurement questionnaire was adapted from the literature. Data collection was performed by five B.Sc. nurses. To assure the quality of the data properly designed data collection instrument and training of data collectors and supervisors was done, the enumerators and the supervisor were given training for three days on procedures, techniques, ways of collecting the data, and monitoring the procedure. Ten percent pretest was done at the Shul public general hospital to check the consistency of the questioner. The collected data were reviewed and checked for completeness by supervisors and principal investigators each week. MUAC was measured by considering the mothers in Frankfurt plane and sideways to measure the left side, arms hanging loosely at the side with the palm facing inward, taken at marked midpoint of upper left arm, a flexible nonstretchable tape should be used, and difference between trainee and trainer should be 0–5 mm.

### 2.6. Independent and Dependent Variables

Nutritional status of pregnant mothers is the outcome variable, and the independent variables were all the sociodemographic characteristics and maternal obstetrical and gynecology history. A brief description of how some of these variables were measured is as follows.

### 2.7. Dependent Variable

The mid-upper arm circumstance values below a cutoff point <23 cm were considered as undernutrition in this study, whereas for the individual in the third-trimester (23 cm and above), it was considered normal [12].

### 2.8. Potential Confounding Factors

Potential confounding variables measured in the study were sociodemographic characteristics and obstetrics and gynecology including the age of mother, marital status, religion, educational background of mothers and household, income, occupation, ethnicity, number of antenatal care visits, type of pregnancy, maternal previous surgery, malaria, parity, iron and folic acid supplementation, marriage at age, hemoglobin level, coffee intake, husband's support, depression, difficulty to access food during the last three months, and history of low birthweight.

### 2.9. Anthropometric Measurement

The anthropometric measurement midupper arm circumstance was taken from individual third-trimester pregnant women.

### 2.10. Data Management and Analyses

After data were entered into Epi-Data 3.1, they were exported to (SPSS) Version 23 for analysis. Binary logistic regression analysis was executed to see the association between independent and outcome variables. All explanatory variables associated with the outcome variable with *p* < 0.25 were entered into multivariable logistic regression analysis, and a significant association was identified based on *p* < 0.05 and AOR with 95% CI.

## 3. Results

### 3.1. Socioeconomic and Demographic Characteristics of Pregnant Women

A total of 840 pregnant women were included in the study with a response rate of 99.5%. The mean age of the pregnant women was 36 (SD + 1.3) years. About 747 (88.9%) pregnant women were married, 322 (38%) of them were pregnant doing agriculture, 280 (33.3%) of mothers were aged from 30 to 34 years, about 772 (91.9%) of the pregnant women were of Tigray ethnicity, and 794 (71.2%) were orthodox in religion. Out of 747 married mothers, 268 (31%) of their husbands had a high school degree, 322 (38.3%) pregnant women were of elementary school, and 280 (33.3%) pregnancy women had a monthly income less than 1000 Ethiopian Birr (Table 1).

### 3.2. Health and Health Care Service Characteristics of Pregnant Women

Out of 840 participating in the study yielding to a response rate of 99.5%, undernutrition was found in 40.6%. About 606 (78.8%) had two-time antenatal care visit, 435 (51.7%) out of pregnant women had wanted type pregnancy, 732 (5178%) had no history of previous surgery, 771 (91.78%) had no history malaria during pregnancy, 596 (70.95%7) had no history of low birthweight, 435 (51.78%) pregnant women had multiple ≥ 5 children, \about 710 (84.5%) pregnant women were in iron and folic acid supplementation during the current pregnancy, 652 (77.6%) pregnant women were married at the age >18 year, 590 (70.2%) pregnant women had a hemoglobin level >11 g/dl, 560 (66.6%) women were daily coffee drinkers, 450 (53.5%) women's husbands were supported them during the current pregnancy, about 650 (77.38%) women had no history of depression, and 598 (71.19%) had difficulty to access food in the last three months (Table 2).

### 3.3. Results of Logistic Regression Analysis

The bivariate analysis showed that malaria, maternal educational status, type of pregnancy, coffee intake, history of low birthweight, hemoglobin, iron and folic acid supplementation, occupation, age of mothers, income, age during marriage, type of pregnancy, and difficulty in food supplementation were crudely associated with 25% level of significance.

Variables associated with adjusted analysis: pregnant women whose occupation is agriculture were more likely [AOR = 2.6, 95% CI: 1.5, 4.5] to have undernutrition than their counterpart, women who had wanted type pregnancy were less likely [AOR = 0.25, 95% CI: 0.14, 0.448] to be undernutritioned than that of planned-pregnancy women; mothers with no malaria infection during pregnancy were less likely [AOR = 0.291, 95%: (0.152, 0.555)] to become undernutritioned than their counterpart; mothers who were coffee drinkers were more likely [AOR = 1.6, 95% CI: 1.04, 2.69] to be undernutritioned than their counterpart; mothers with hemoglobin < 11 g/dl were more likely [AOR = 4.9, 95% CI: 3.09, 7.8] to be undernutritioned than their counterpart (Table 3).

## 4. Discussion

In this study finding, the magnitude of undernutrition in pregnant women was 40.6% with 95% CI (38.93% and 42.27%), which is higher than that in the study conducted in Alamata (22.3%), Bangladesh (23.5%) [13], Amhara Gonder (14.4%) [14], eastern Ethiopia (23.3%) [15], southern Ethiopia (20.9%) [16], Sri Lanka [17], and southern Ethiopia (31.4%) with MUAC < 22 [18] of the participated pregnant women who were undernutritioned. This discrepancy may be because of the study setting and the cutoff point that was used between the studies and the term of the study participants. However, this study was almost similar to the study performed in Oromia Region (38.3%) [19]. Whereas compared with our finding, a lower prevalence of undernutrition was reported in Alta Choko, southern Ethiopia (71.1%) [20] and Ardal country (51.3%) [21]. This may be due to the MUAC cutoff point <19–22 cm that they used, sample size difference, and sociodemographic difference.

Mothers whose occupation is agriculture were 2.6 times more likely [AOR = 2.6, 95% CI: 1.5, 4.5] to have undernutrition than those who are housewives. This study is similar to the study performed in pregnant women living in Bangladesh doing agriculture who were more undernutritioned and to the other studies which were conducted in Ethiopia [13, 16, 22]. This is because mothers in low-income countries are spending their time to do agriculture than caring for themselves, and the working intensity might also be hard for pregnant women.

In this study, mothers with wanted type pregnancy were 75% less likely [AOR = 0.25, 95% CI: 0.14, 0.448] to became undernutritioned than those with unwanted pregnancy. This finding is similar to the evidence from low- and middle-income countries which indicated that unwanted pregnancy can affect maternal nutrition, health service, and child nutrition [23, 24]. This is because mothers with wanted pregnancy type have planned programs on how to feed themselves and prepare to have diversified food types in their homes.

Pregnancy mothers with no malaria during pregnancy were 70.09% less likely [AOR = 0.291, 95%: (0.152, 0.555)] to become undernutritioned than those having malaria during pregnancy. This finding was similar to that of the study performed in Nigeria, in which it was found that malaria can cause anemia, another study performed in Alamata Tigray, a study performed in Amazonian Region [14, 16, 25–27], and others. This is because malaria is one of the proximal factors that cause undernutrition in pregnancy.

Mothers consuming coffee was 1.6 times more likely [AOR = 1.6, 95% CI: 1.04, 2.69] to be undernutritioned than those in the counterpart. This finding was similar to that of the study performed in Costa Rica, Ethiopia, and also that of other studies [28–30]. This because coffee intake affects iron bioavailability, and due to its potency as an inhibitor of absorption, it is likely to aggravate anemia at times of increased physiological need or when dietary iron intake is precarious and can compete with other nutrients [31]. Drinking coffee can cause nutrient depletion of important nutrients, such as vitamin B6, and interfere with nutrient absorption of essential minerals, including calcium, iron, magnesium, and vitamin B.

Mothers with hemoglobin < 11 g/dl were 4.9 times more likely [AOR = 4.9, 95% CI: 3.09, 7.8] to have undernutrition than their counterparts. This finding was similar to that of the study performed in Wonde Genetin, southern Ethiopia, Nigeria and study done in Reft valley [4, 25, 26, 32, 33]. Women who were anemic were also more likely to have a low MUAC. A study demonstrated that an MUAC measurement above 23 cm reduced odds of anemia by 0.41.

## 5. Conclusion and Recommendation

Above one-third of pregnant mothers were undernutritioned in this study, it was found to be higher than that in other similar studies; it should be considered as a major public health problem in this study area. Our study also identified malaria during pregnancy, occupation, coffee intake, hemoglobin level, and type of pregnancy were factors associated with pregnant mothers' undernutrition, so healthcare providers and health organizations should give special focuses on these factors. Multisectoral collaboration and coordination between national and international organizations will also be needed for prevention. Counseling services for pregnant mothers might also help to improve their nutritional status. The mothers shall also be counseled about the effect of frequent coffee consumption to their fetus and their health.

### 5.1. Limitation of This Study

The cross-sectional nature of the study might affect the establishment of a causal relationship between identified risk factors and undernutrition; this study is an institution-based study, and other health services were not included. This study failed to incorporate the dietary diversity score as a risk factor of undernutrition which might have also introduced a residual confounding problem.

## Figures and Tables

**Table 1:** Socioeconomic and demographic characteristics of pregnant women in public general hospitals of Tigray Ethiopia 2018 (*n* = 840).

Variable	Category	Frequency	Percent
Marital status	Divorced	93	11.07
	Married	747	88.9

Occupation	Housewife	142	16.9
	Agriculture	322	38.3
	Government employee	268	31.9
	Nongovernment employee	110	13.09
	Housewife	142	16.9

Religion	Orthodox	794	94.5
	Muslim	46	5.47

Mother's age	15–19	48	5.7
	20–24	36	4.28
	25–29	42	5
	30–34	280	33.3
	35–39	223	26.5
	40–44	154	18.3
	45–49	57	6.78

Husband's education	No education	167	19.88
	Elementary	231	27.5
	High school	268	31.9
	Preparatory school	58	6.9
	College and above	116	13.8

Ethnicity	Tigray	772	91.9
	Others	68	8

Mother's education	No education	142	16.9
	Elementary	322	38.3
	High school	268	31.9
	College and above	108	12.8

Income (Ethiopian Birr)	Less then 1000	280	33.3
	1001–2000	225	26.78
	2001–3000	246	29.2
	Above 3001	89	10.59

**Table 2:** Frequency distribution of health and health-related variables among pregnant women in public general hospitals of Tigray, Ethiopia 2018 (*n* = 840).

Variable	Category	Frequency	Percent
Type of pregnancy	Unwanted	159	18.9
	Unplanned	146	17.38
	Wanted	435	51.78

Maternal previous surgery	Yes	37	4.8
	No	732	95.2

Malaria	Yes	69	8.2
	No	771	91.78

How many ANCs?	One	25	3.3
	Two	606	78.8
	Three and above	138	17.9

Coffee intake	Yes	676	80.4
	No	164	19.5

History of low birthweight	Yes	245	29.16
	No	596	70.95

Parity	Primipara	159	18.9
	2–4 children	246	29.28
	Multiple ≥ 5	435	51.78

Iron and folic acid supplementation	Yes	710	84.5
	No	130	15.47

Age at marriage	<18 year	188	22.38
	>18 year	652	77.6

Hemoglobin < 11 g/dl	Yes	250	29.76
	No	590	70.2

MUAC < 23 cm	>23 cm	499	59.4
	<23 cm	341	40.59

Husband's support	Always	450	53.57
	Sometimes	152	18.09
	Never	238	28.3

Depression	Yes	90	10.7
	No	650	77.38

Difficulty to access food in the last three months	Yes	142	16.9
	No	598	71.19

**Table 3:** Factors associated with undernutrition in pregnant women in public general hospitals of northern Ethiopia 2018 (*n* = 840).

Variables	Category	Instrument		COR 95% CI	AOR 95% CI
		Yes	No		
Difficulty in food supplementation during the last 3 months	No	469	303	0.510 (0.309, 0.841)	0.716 (0.374, 1.369)
	Yes	30	38	1	1

Age of the mother	15–19	28	20	1	1
	20–24	24	12	0.700 (0.285, 1.721)	0.774 (0.239, 2.504)
	25–29	26	16	0.862 (0.369, 2.009)	1.798 (0.596, 5.426)
	30–34	178	102	0.802 (0.430, 1.496)	1.219 (0.514, 2.891)
	35–39	111	112	1.413 (0.752, 2.655)	2.093 (0.857, 5.109)
	40–44	102	52	0.714 (0.367, 1.386)	1.260 (0.510, 3.114)
	45–49	30	27	1.260 (0.581, 2.733)	1.497 (0.516, 4.34)

Occupation	Housewife	89	53	1	1
	Agriculture	166	156	1.578 (1.053, 2.364)	2.643 (1.548, 4.513)^*∗∗*^
	Government employee	180	88	0.821 (0.537, 1.256)	1.576 (0.848, 2.927)
	Nongovernment employee	64	44	1.154 (0.691, 1.928)	2.295 (0.971, 5.42)

Type of pregnancy	Unwanted	36	123	1	1
	Planned	115	131	0.333 (0.213, 0.522)	0.777 (0.447, 1.350)
	Wanted	348	87	0.073 (0.047, 0.114)	0.253 (0.143, 0.448)^*∗∗*^

Mother's educational status	No education	140	27	0.396 (0.225, 0.697)	0.617 (0.319, 1.193)
	Elementary	153	78	1.046 (0.651, 1.681)	0.988 (0.558, 1.74)
	High school	103	165	3.288 (2.077, 5.205)	1.649 (0.941, 2.89)
	Preparatory school	25	33	2.709 (1.417, 5.182)	1.048 (0.452, 2.427)
	College and above	78	38	1	1

Malaria status	No	479	292	0.249 (0.145, 0.427)	0.291 (0.152, 0.555)^*∗∗*^
	Yes	20	49	1	1

Coffee intake	Yes	398	278	1.120 (0.789, 1.589)	1.678 (1.046, 2.690)^*∗*^
	No	101	63	1	1

History of low birthweight and preterm	Yes	148	97	0.943 (0.696, 1.277)	1.146 (0.643, 2.043)
	No	351	244	1	1

Age at marriage	<18 years	144	44	0.365 (0.252, 0.529)	0.685 (0.383, 1.226)
	>18 years	355	297	1	1

Hemoglobin < 11 g/dl	Yes	59	191	9.496 (6.720, 13.4)	4.943 (3.097, 7.888)^*∗∗*^
	No	440	150	1	1

Iron and folic acid supplementation	Yes	398	312	2.730 (1.761, 4.2)	1.202 (0.613, 2.36)
	No	101	29	1	1

Income	Less than 1000	190	90	1	1
	1001–2000	129	96	1.571 (1.091, 2.261)	1.344 (0.842, 2.146)
	2001–3000	137	109	1.680 (1.177, 2.396)	1.288 (0.821, 2.022)
	Above 3001	43	46	2.258 (1.39, 3.6)	1.126 (0.582, 2.18)

^*∗∗*^ *p* ≤ 0.001; ^*∗*^*p* < 0.05.

## Data Availability

The complete dataset used and/or analyzed during the current study are available from the corresponding author and can be accessed upon reasonable request.

## Abbreviations

ANC: Antenatal care
AOR: Adjusted odds ratio
CI: Confidence interval
COR: Crude odds ratio
CS: Caesarean section
cm: Centimeters
MUAC: Mid-upper arm circumference.
